# The Epidemiology of PCR-Confirmed Cutaneous Leishmaniasis in Israel: A Nationwide Study

**DOI:** 10.3390/microorganisms12101950

**Published:** 2024-09-26

**Authors:** Dror Avni, Michal Solomon, Merav Strauss, Orli Sagi, Violeta Temper, Ayelet Michael-Gayego, Tal Meningher, Emily Avitan-Hersh, Moran Szwarcwort-Cohen, Jacob Moran-Gilad, Ayelet Ollech, Eli Schwartz

**Affiliations:** 1 Laboratory for Tropical Diseases Research, Chaim Sheba Medical Center, Tel Hashomer, Ramat-Gan 52621, Israel; droravni@msn.com (D.A.); tal.meningher@sheba.health.gov.il (T.M.); elischwa@tauex.tau.ac.il (E.S.); 2 The Faculty of Medicine, Tel Aviv University, Tel Aviv 6997801, Israel; 3 Department of Dermatology, Chaim Sheba Medical Center, Tel Hashomer 52621, Israel; 4 Molecular Microbiology Laboratory, HaEmek Medical Center, Afula 183111, Israel; merav.strauss@gmail.com; 5 Clinical Parasitology, Soroka University Medical Center and the Faculty of Health Sciences, Ben-Gurion University, Beer-Sheva 84101, Israel; orlisa@clalit.org.il; 6 The Faculty of Health Sciences, Ben-Gurion University, Beer-Sheva 84101, Israel; 7 Hadassah Medical Center, Jerusalem 91120, Israel; vtemper@hadassah.org.il (V.T.); ayeletg@hadassah.org.il (A.M.-G.); jacobmg@hadassah.org.il (J.M.-G.); 8 Departments of Dermatology, Rambam Health Care Campus and Technion Faculty of Medicine, Haifa 31096, Israel; e_avitan@rambam.health.gov.il (E.A.-H.); m_szwarcwort@rambam.health.gov.il (M.S.-C.); 9 Microbiology, Rambam Health Care Campus, Haifa 31096, Israel; 10 Pediatric Dermatology Service, Shaare Zedek Medical Center, Jerusalem 9103102, Israel; ayeletollech@gmail.com; 11 The Faculty of Medicine, Hebrew University of Jerusalem, Jerusalem 91120, Israel; 12 The Institute of Geographic Medicine and Tropical Diseases, Sheba Medical Center, Tel Hashomer 52621, Israel

**Keywords:** cutaneous leishmaniasis, visceral leishmaniasis, *L.* (L.) *major*, *L.* (L.) *tropica*, *L.* (L.) *infantum*, *L.* (V.) *braziliensis*

## Abstract

Background: Leishmaniasis, mainly cutaneous leishmaniasis (CL), is endemic in Israel. In recent years, the diagnosis of leishmaniasis has transitioned to a molecular diagnosis. Objective: To summarize all cases of leishmaniasis and the identified species seen in Israel based on molecular diagnosis. Methods: A retrospective study was performed of patients diagnosed with leishmaniasis between January 2017 and December 2022. All five medical centers in Israel in which Leishmania diagnosis is performed were included: Soroka, HaEmek, Hadassah, Rambam, and Sheba, all utilized molecular diagnostic methods. Data on the annual number of cases, species, age, and gender were retrieved. Results: During the years 2017–2022, a total of 4168 cases of leishmaniasis were diagnosed, which corresponds with ~7/100,000 inhabitants. *L.* (L.) *major* and *L.* (L.) *tropica* accounted for 84% and 14%, respectively. During the years 2020–2021, *L.* (L.) *infantum* emerged as a new form of cutaneous disease [2.7% of cases during this period]. Visceral *L.* (L.) *infantum* was found in five cases. Imported New World leishmaniasis accounted for 1% of the cases. *L.* (L.) *major* affected more males (67%) while *L.* (L.) *tropica* commonly affected more children and caused more facial lesions. Conclusions: The mean annual number of cases during these years is ~700. The dominant species is *L.* (L.) *major.* Since 2020, cutaneous *L.* (L.) *infantum* is an emerging infection in Israel.

## 1. Introduction

Leishmania is a protozoan that causes a wide variety of diseases. The clinical presentation includes cutaneous leishmaniasis (CL), visceral leishmaniasis (VL), or mucocutaneous leishmaniasis (MCL) and depends mainly on the infecting species [1]. In Israel, Old World CL is attributed to *Leishmania major* (*L.* (L.) *major*) [2,3] and *Leishmania tropica* (*L.* (L.) *tropica*) [4]. VL, mainly caused by *L.* (L.) *infantum*, is also reported in Israel, albeit in very small numbers [5]. Previous data (2000–2022) from the Israel Ministry of Health’s Epidemiology Department indicated a CL incidence rate in Israel of 1–4 cases per 100,000 population. Over the past two decades, there has been an upsurge in travel to South and Central America among young Israeli adults, increasing potential exposure and importation of tropical diseases, including New World leishmaniasis [4,6]. New World cutaneous leishmaniasis, endemic in some parts of the Americas, is caused by the *Leishmania* (*Viannia*) subgenus and the *Leishmania* (*Leishmania*) *mexicana* subgenus. Infection with the sub-genus *Viannia*, particularly *Leishmania* (*Viannia*) *braziliensis* (*L.* (V.) *braziliensis*), results in CL that tends to be persistent. In addition, CL may later progress to MCL [7,8].

In past years, the diagnosis of leishmaniasis, particularly cutaneous leishmaniasis (CL), was commonly performed by scraping the skin lesion and applying Wright-Giemsa stains to the sample [9]. This staining technique allows for the identification of the amastigote form of the Leishmania protozoa, which appear as distinctive blue, round, or oval bodies known as Leishman–Donovan bodies. Under the microscope, amastigotes appear as small, round or oval bodies (2–4 μm) within macrophages. They have a darkly stained nucleus, a smaller kinetoplast, and bluish cytoplasm, often resembling a “swarm of bees” when clustered [9].

This traditional method, although effective, has been increasingly replaced by more advanced molecular diagnostic techniques in recent years, which offer higher sensitivity and specificity [10].

In the last decade, the diagnosis of leishmaniasis in Israel has transitioned to molecular methods, owing to their high sensitivity and capacity for species-specific identification. Currently, Leishmania diagnosis is conducted in five medical centers across Israel, all using molecular diagnostics. While leishmaniasis is a notifiable disease in Israel, it is underreported, and therefore accurate national surveillance data are lacking.

In the present study, we report on the epidemiology of leishmaniasis in Israel with emphasis on the infecting species, based on molecular diagnosis, during a six-year period between January 2017 through December 2022. 

## 2. Patients & Methods

**Figure 1:**
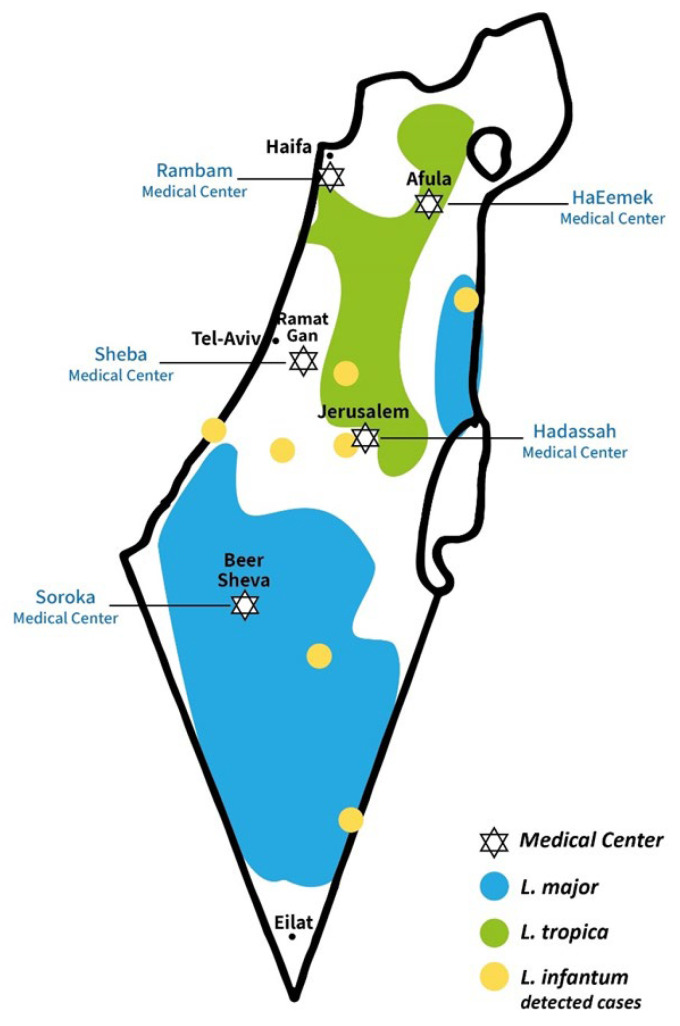
A Map depicting leishmania-endemic regions and medical centers offering molecular diagnosis in Israel. The map is based on Jaffe CL et al.’s Leishmaniasis in Israel and the Palestinian Authority. Trends Parasitol. 2004 2 with our modification according to accumulated data. *L.* (L.) *infantum* cases are based on our previous data [11].

Study sites: The medical centers of Sheba (central Israel), HaEmek (North-east Israel), Rambam (north-west), Hadassah (Jerusalem area), and Soroka (southern Israel) are the sites in Israel performing routine laboratory diagnosis of leishmaniasis (Figure 1). We collected all PCR-confirmed patient-unique cases reported between the years 2017 and 2022. Information regarding the annual number of cases, patient age (children < 18 years old; adults > 18 years old), and sex was retrieved from all participating medical centers. Information regarding causative species was retrieved from four centers since one center (Rambam Medical Center) did not perform routine speciation.

Information regarding the geographic location of disease acquisition and the anatomical sites of the infection was recorded in a subset of cases where data were available. Samples of VL (visceral leishmaniasis) were taken from blood.

The institutional review board of Sheba Medical Center approved the study (protocol approval no. 7274-09).

### 2.1. Molecular Diagnosis in Sheba Medical Center

#### 2.1.1. DNA Extraction

Tissue specimens were taken from the border of suspected skin lesions. The edges of the lesion were cut with a sterile scalpel. The tissue specimens were spread on 3 mm filter paper (Watman No. 3) and air-dried. The paper was cut and inserted into a 1.5 mL test tube. The DNA was extracted from the dried blood spots using the QIAamp DNA Mini Kit (QIAGEN catalog number 51304) according to the manufacturer’s instructions written in the Protocol: Isolation of Genomic DNA from Dried Blood Spots.

#### 2.1.2. PCR-RFLP Analysis

The extracted DNA was subjected to PCR amplification using the primers to ITS1 with MyTaq HS Red Mix, 2x (Meridian Life Science, Inc., Memphis, TN 38134-5611, USA) [12]. The ITS1 region, located between the 18S and 5.8S ribosomal RNA genes, is frequently used because it provides high variability between different species of Leishmania, making it suitable for species differentiation. Next, the amplification produced was subjected to enzymatic digestion with HaeIII (New England BioLabs catalog number R0108). The diagnoses of New World *L.* (V.) *lainsoni*, *L.* (V.) *braziliensis*, *L. guyanensis*, and *L. panamensis* were based on amplification of the *hsp70* gene and digestion with BccI or HaeIII as shown in Montalvo, AM. et al. [13]. The digested products were subjected to electrophoresis in a 4% agarose gel composed of 1% MetaPhor^TM^ Agarose (Lonza catalog number 50181) and 3% SeaKem^®^ LE Agarose (Lonza catalog number 50004). DNA fragments were visualized under ultraviolet light.

### 2.2. Molecular Diagnosis at Soroka and HaEmek Medical Centers

Samples were taken from suspected skin lesions using a swab applicator placed into a tube containing transfer media (FLOQswabs; Copan, Murrieta, CA 92010, USA) [10]. Nucleic acid extraction (500 μL of the into 50 μL of elution solution) was performed using NucliSense EasyMag (bioMerieux, Marcy l’Etoile, France). Starlet extraction automate (SeeGene) is implemented in both HaEmek and Soroka medical centers. The extracted DNA was amplified by multiplex PCR combining a set of forward and reverse primers with five different probes for detection of leishmania spp., *Leishmania major*, *Leishmania tropica*, *Leishmania braziliensis*, and *Leishmania infantum/donovani* [10].

A high-resolution melting (HRM) assay was employed to detect the infection and determine the species involved.

### 2.3. Molecular Diagnosis in Hadassah Medical Center

Fresh skin samples were obtained in the laboratory by scraping the patients’ lesions. [12] DNA was extracted using the NucliSENS easyMag DNA Purification Kit (BioMerieux, Lyon, France). Molecular detection of the ITS gene target was performed by real-time PCR using SYBR chemistry on a Rotorgene instrument (QIAGEN, Hilden, Germany). Analysis was performed using melting curve analysis according to the infecting species. Sanger sequencing of the PCR product was performed for validation in selected cases.

### 2.4. Molecular Diagnosis at Rambam Medical Center

Tissue specimens were taken from suspected skin lesions. The lesions were pierced with a 21G needle, and fresh blood was extracted using a UTM^®^ Universal Transport Medium™ swab (COPAN Diagnostics Inc., Murrieta, CA 92562, USA) [12].

The DNA was extracted using MagLead Precision System Science Co. (PSS), Chiba, Japan, according to the manufacturer’s instructions. PCR was performed using the Clonit© leishmania spp. Kit (RT-63) (CLONIT srl, 20081 Abbiategrasso, Milan, Italy). Species analysis was not performed.

### 2.5. Statistical Analysis

Statistical package for the social sciences (SPSS) version 23.0 for Windows software was used for the data entry and analysis. Continuous variables were expressed as medians and interquartile ranges (IQR) and categorical variables as percentages.

Fischer’s exact test (two-tailed) was used to compute the *p*-value in the prevalence assessment. A *p*-value < 0.05 was considered significant.

All statistical tests and graphing were performed using GraphPad Prism version 5.01 August 2007 for Windows, GraphPad Software, San Diego, CA, USA, www.graphpad.com (accessed on 20 August 2024).

## 3. Results

**Figure 2:**
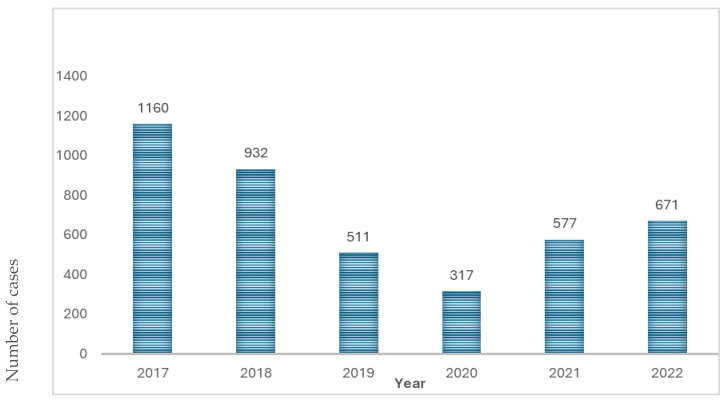
Annual number of leishmania cases between the years 2017–2022.

**Figure 3:**
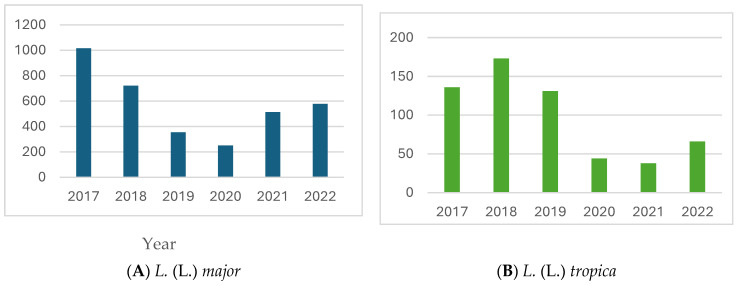
Annual number of patients diagnosed with *Leishmania major* and *Leishmania tropica*.

**Figure 4:**
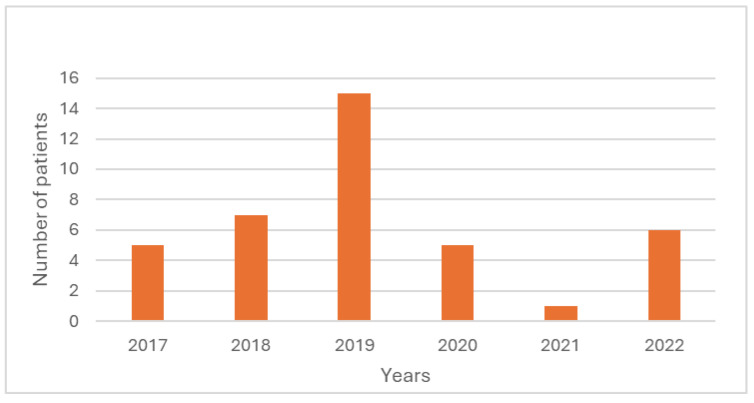
Total number of patients diagnosed with *Leishmania braziliensis* between the years 2017–2022.

Between 2017 and 2022, a total of 4168 cases of leishmaniasis were reported by the five sites in Israel. The annual number of reported cases varied from 1160 in 2017 to the nadir of 317 cases in 2020, with a subsequent increase in 2021 and 2022 (Figure 2). This trend in case numbers during 2019–2020 was observed across all tested species, as illustrated in Figure 3 for *L.* (L.) *major* and *L.* (L.) *tropica*, while in the South American species, the decline was during 2020–2021, correlating with the COVID-19 pandemic-related travel restriction (Figure 4).

The infections were acquired in different regions of Israel, including the southern part (Negev and Arava regions), which is known to be endemic to *Leishmania major*, and the central (eastern to Tel Aviv region) and Galilee regions, which are known to be endemic to *Leishmania tropica* (Figure 1). New occurrences of leishmania were detected in at least three urban areas (Tiberias, Yeruham, and Ofakim), underscoring the evolving epidemiological landscape of the disease.

The distribution of leishmania species diagnosed during this period is based on four reporting centers (since one center does not perform species-specific diagnosis) and comprises 98.3% of cases (4100/4168). The total number and species of leishmania cases are presented in Table 1. Eighty-four percent of the patients tested positive for *L.* (L.) *major*, and 14.4% tested positive to *L.* (L.) *tropica*. During the years 2020–2021, *L.* (L.) *infantum* emerged as a new form of cutaneous disease that accounted for 22 cases (equivalent to 2.7% of the cases during this two-year period). In addition, five cases of visceral *L.* (L.) *infantum* were also diagnosed. All except one were acquired in Israel.

Of the 4033 leishmania cases for which data on patient sex were available, 2648 (65.6%) were males and 1385 (34.3%) were females. Demographic and clinical data are shown in Table 2. Notably, among the 4100 leishmania patients for whom the information was available, the majority of those infected with *L.* (L.) *major* were males, comprising 65.7% of the patient’s vs. 57.3% in *L.* (L.) *tropica* patients (*p* < 0.0001). Additionally, children (under the age of 18) accounted for 19.9% of *L.* (L.) *major* patients vs. 31.0% among *L.* (L.) *tropica* (*p* < 0.0001).

The body-site of the infection is important, and we focused on facial skin lesions. Information on the location of skin lesions was available in only three medical centers. Comparing the facial lesions between the two common endemic species in Israel showed that facial lesions were significantly more common in *L.* (L.) *tropica*, accounting for 34.0% (116 out of 341 patients) vs. only 15.9% (76 out of 495 patients) of the *L.* (L.) *major* cases (*p* < 0.0001) (Table 2). Imported New World leishmaniasis accounted for 0.95% (39 cases) of the total cases. In this cohort, a sharp decline was noticed during 2020–2021, correlating with the COVID-19 travel restrictions (Figure 4). Most of these cases (26/39 = 66%) were seen at Sheba Medical Center, and more detailed information was available for them. Based on this information, all leishmania patients except one acquired the disease in South America, mostly in the Amazon region of Bolivia (*N* = 17), particularly at Madidi National Park. Almost all these cases were due to either *L.* (V.) *braziliensis*/*L. guyanensis* (differentiation between *L.* (V.) *braziliensis* and *L. guyanensis* was not performed). In one exceptional case, from Bolivia, *L.* (V.) *lainsoni* was detected. One case was acquired in Central America (Costa Rica), in which the species was *L. panamensis*.

## 4. Discussion

Leishmaniasis, mainly CL, is endemic to Israel and is increasing in frequency and regional distribution [14]. Historically, CL in Israel has been attributed almost exclusively to *Leishmania major* (*L.* (L.) *major*) [3]. *L.* (L.) *major* exists mainly in the southern part of Israel (Negev and Arava, Figure 1). However, over the last two decades, CL due to *L.* (L.) *tropica* has emerged, and it is increasingly reported in several other regions of Israel, including: greater Jerusalem and northern and eastern Israel [15]. VL due to *L.* (L.) *infantum* is known to exist in Israel, but reported infrequently in humans [16]. *L.* (L.) *infantum* CL, which is known to exist in the Middle East region, was not diagnosed previously in Israel. However, a first case was reported in Israel in 2016 [17]. and recently we reported eight more cases in Israel [11].

In this study, we report all the cases of PCR-confirmed leishmaniasis and leishmania species identified in Israel.

The advantage of molecular leishmania diagnosis is its speciation ability in addition to the improved sensitivity of PCR diagnosis as compared to microscopic diagnosis as well as robustness with respect to operator dependency. In various studies comparing the sensitivity of Giemsa staining and PCR, Giemsa staining was found to be less sensitive by 10–50% [18,19,20]. Similarly, at Soroka Medical Center, a comparison between Giemsa staining and qPCR showed that qPCR was approximately 15% more sensitive [10]. A comparison between ITS1 PCR-RFLP and Giemsa staining in Sheba Medical Center has also shown that PCR was more sensitive by ~20% [10,20].

Our results show that the mean annual number of cases that were diagnosed in Israel in the past six years is approximately 700 new cases per year, with the highest number, 1160 patients, seen in 2017. These figures result in an annual incidence rate of ~7 cases per 100,000 people. In Israel, leishmaniasis is a notifiable disease, but it is based on passive reporting only. Previous data (2017–2022) from the Israel Ministry of Health’s Epidemiology department showed a much lower (about half) incidence rate of 3 per 100,000 people. This difference could be due to variations in leishmania activity, but it could also be due to the advantages of molecular diagnosis, which was not used until later. However, epidemiological reports indicate that there were only 1438 cases of cutaneous leishmaniasis in the years 2017 to 2022 (our study period), whereas our cohort included more than 4000 cases in the same time frame. This difference is also reflected in the incidence rate of 1.94 reported to WHO from 2017 to 2020 [21], highlighting the extent of underreporting of leishmania cases by passive data collection.

Our annual incidence results of about 7/100,000 inhabitants of leishmaniasis in Israel are in the same order of magnitude as reported by neighboring countries such as Arab Saudi Arabia (5–10), Lebanon (3.7), and Jordan (1.1) per 100,000 [22], which are about 10 times less compared to northern African countries (such as Tunisia, Libya, Algeria, and Morocco). In the affected EU countries in southern Europe the incidence is much lower and is about 0.27 in France and 0.40 per 100,000 inhabitants in Spain [21]. Our study is most likely the only nationwide Israeli study conducted in Israel that relies solely on molecular diagnosis.

The dominant species causing leishmaniasis in Israel is *L.* (L.) *major*, which accounts for 84% of cases, followed by *L.* (L.) *tropica,* which accounts for 14.4% of cases. These two species belong to the Old World leishmaniasis, and, as such, they were lumped together in previous reports. A possible explanation for this is that the diagnosis was based on microscopy, which cannot distinguish between the two. However, there are significant differences between these two species, both in reservoirs and their clinical perspectives. The reservoirs of these two leishmania species are different. *L. major*’s reservoir includes *Psammomys obesus* and *Meriones crassus* [3], while *L.* (L.) *tropica*’s reservoir includes mainly rock hyraxes (Procavia capensis) [23], and there are different approaches on how to control these hosts. From a clinical perspective, CL caused by *L.* (L.) *tropica* heals more slowly, is relatively resistant to treatment, and tends to recur more often in comparison to *L.* (L.) *major* [24,25]. In our current study, we found several other differences between these two leishmania species: *L.* (L.) *major* was more common in males, while *L.* (L.) *tropica* was more evenly distributed in both genders. *L.* (L.) *tropica*. was significantly more common in children (in a third of the cases—see Table 2), and importantly, *L.* (L.) *tropica* was associated with a higher incidence of cutaneous facial lesions in comparison to *L.* (L.) *major*. (Table 2) [26]. *L.* (L.) *tropica’s tendency* to affect the face was also reported in northern Africa [27,28]. Overall, it is important to emphasize that further reports on the epidemiology and clinical aspects of leishmania should be based on species-specific diagnoses. To ensure more accurate and informative data, this approach is preferable to using the generalized terms ‘Old World leishmaniasis’ and ‘New World leishmaniasis’.

New World (NW) leishmaniasis is encountered in Israel as a disease imported by Israeli travelers returning from Latin America. As such, the decline in imported cases between the years 2020–2021 (Figure 4) is most likely related to the COVID-19 pandemic travel restrictions.

The vast majority of NW leishmaniasis cases were of *L. v. braziliensis* complex [6] and were acquired in South America, mainly in the Amazon basin of Bolivia. Over the 6-year period of this study, we diagnosed 39 cases (~1% of all our leishmania cases), which implies that laboratories performing leishmania molecular diagnosis should ideally be equipped with the ability to detect New World leishmania species as well. Most of the New World leishmaniasis cases were seen at Sheba Medical Center (66%), which is the main tropical disease referral center in Israel. From the data we have analyzed, there was only one case of *L. panamensis* from Central America. The remaining cases were predominantly from South America, involving *L.* (V.) *braziliensis/guyanensis*, except for one instance of *L.* (V.) *lainsoni*, which was acquired in Bolivia.

From our experience, most New World leishmaniasis cases presented with cutaneous manifestations only, but approximately 14% of cases had mucosal involvement as well [8].

*L.* (L.) *infantum* is the species known to cause VL, a fatal disease, which is also endemic in the Middle East—including Israel. The number of cases in Israel is usually low [16], and in the current study we have accounted for five cases over a 6-year period. The interesting phenomenon is the emergence of *L.* (L.) *infantum*-related CL. Although it is known to exist in the Middle East region, in Saudi Arabia, Turkey, and Yemen [11], for example, it had not been diagnosed in Israel in the past. It was first reported in 2016, and ever since, increasing numbers of cases have emerged [11]. In this study, specifically, we report 24 cases diagnosed over the study’s last two years (2021–2). However, without molecular diagnostics, the detection of CL due to *L.* (L.) *infantum* is very limited. The precise mechanism behind the phenomenon where a solitary species such as *L.* (L.) *infantum* can result in either a mild CL disease or a potentially fatal VL is still not fully comprehended. Factors involving both the host and the parasite, have been suggested as potential causes [29,30] but these hypotheses require further investigation.

## 5. Limitations of Our Study

This is the most comprehensive study on the incidence and the etiology of leishmania circulating in Israel, based on molecular methods. However, despite the fact that the cases reported herein are based on laboratory records from all centers in Israel, they might still represent an under-estimation of the true incidence, as many patients do not seek medical care or are not referred for diagnostic procedures (especially those who live in endemic foci and rely solely on clinical diagnosis), despite molecular testing being available under the national health insurance scheme. Thus, our estimation of an incidence of ~7/100,000 population might be well below the true incidence and deserve a separate study. Another limitation is the utilization of diverse laboratory assays by participating laboratories, which could contribute to the variation in PCR test performance. Furthermore, one center lacked species data, and two centers lacked demographic data, but since they accounted for <5% of all cases, they probably could not skew the data significantly. Data concerning the likely exposure sites of patients was not available for most cases, a fact that limited our ability to perform a temporo-spatial analysis of CL trends. Another limitation of the study is that the age data were collected only in two categories: below and above 18 years old.

Finally, the study period included the years of the COVID-19 pandemic, which may have affected case numbers due to changes in daily activities and domestic tourism.

## 6. Conclusions

The mean annual number of cases of leishmaniasis diagnosed in Israel over the past 6 years has been ~700 (approximately seven cases per 100,000 inhabitants). There was a significant decrease in the number of cases beginning in 2019 and through 2020, which cannot be solely attributed to the COVID-19 isolation period but rather to variation in leishmania activity.

The most prevalent species in Israel is *L.* (L.) *major*, followed by *L.* (L.) *tropica. L.* (L.) *tropica* most commonly affects children and has a predominant facial distribution. Cases of New-World leishmaniasis, mainly *L. v. braziliensis*, are typically imported to Israel by returning travelers, and therefore medical practitioners should be acquainted with this infection, including its potential mucosal involvement. Since 2020, cutaneous *L.* (L.) *infantum* appears to be an emerging cause of CL in Israel.

## Figures and Tables

**Table 1:** Total number and species of leishmania cases. ^a^ One center was excluded since a species-specific diagnosis was not performed. Their cases account for 1.6% of all cases. ^b^ Of the 27 cases, 22 were classified as CL (cutaneous leishmaniasis), and 5 were identified as VL (visceral leishmaniasis). ^c^ All the cases were from the *Leishmania braziliensis* complex, including *L. braziliensis* and *L. guyanensis*, without further differentiation between the two. Additionally, one case was reported as *L. lainsoni* and another as *L. panamensis*.

Hospital	Total	*L.* (L.) *major*	*L.* (L.) *tropica*	*L.* (L.) *infantum* ^b^	*New World CL* ^c^
Soroka Medical Center	2995 (73%)	2810 (81.6%)	171 (29%)	6 (22%)	8 (20%)
Sheba Medical Center	605 (14.7%)	400 (11.6%)	162 (27.5%)	17 (63%)	26 (67%)
HaEmek Medical Center	283 (7%)	160 (4.6%)	118 (20%)	3 (11%)	2 (5%)
Hadassah Medical Center	217 (5.3%)	76 (2.2%)	137 (23.5%)	1 (4%)	3 (8%)
Total number ^a^	4100	3446	588	27	39
Total %	100%	84%	14.4%	0.65%	0.95%

**Table 2:** Characteristics of patients with *L.* (L.) *major* vs. *L.* (L.) *tropica*.

	*L.* (L.) *major* *N* = 3391	*L.* (L.) *tropica* *N* = 507	*p* Value
Males/Females %	69.3/30.7%	53.8/46.2%	<0.0001
Children (<18 years) Males/Females %	14.2% 55.5/44.5	36.4% 47/53	<0.0001 0.056
Body lesion sites (*N* = 836)			
	***L.* (L.) *major*** ***N* = 495**	***L.* (L.) *tropica*** ***N* = 341**	
Facial lesion %	15.9%	34.0%	<0.0001

## Data Availability

Data is contained within the article. The original contributions presented in the study are included in the article, further inquiries can be directed to the corresponding author/s.

## Abbreviations

CL	Cutaneous leishmaniasis
VL	visceral leishmaniasis
MCL	mucocutaneous leishmaniasis
*L.* (L.) *major*	*Leishmania major*
*L.* (L.) *tropica*	*Leishmania tropica*
*L.* (L.) *infantum*	*Leishmania infantum*
*L.* (V.) *Braziliensis*	*Leishmania viannia braziliensis*
